# Important factors determining the nanoscale tracking precision of dynamic microtubule ends

**DOI:** 10.1111/jmi.12316

**Published:** 2016-01-19

**Authors:** G. BOHNER, N. GUSTAFSSON, N.I. CADE, S.P. MAURER, L.D. GRIFFIN, T. SURREY

**Affiliations:** ^1^ The Francis Crick InstituteLincoln's Inn Fields LaboratoryLondonU.K; ^2^ Centre for Mathematics and Physics in Life Sciences and Experimental Biology (CoMPLEX)University College LondonLondonU.K; ^3^ Present address: Gatsby Computational Neuroscience UnitUniversity College LondonLondonU.K; ^4^ Present address: MRC Laboratory for Molecular Cell BiologyUniversity College LondonLondonU.K; ^5^ Present address: Centre for Genomic Regulation (CRG)Dr. Aiguader 8808003BarcelonaSpain

**Keywords:** Image processing, image simulation, microtubule, TIRF microscopy, tracking

## Abstract

Tracking dynamic microtubule ends in fluorescence microscopy movies provides insight into the statistical properties of microtubule dynamics and is vital for further analysis that requires knowledge of the trajectories of the microtubule ends. Here we analyse the performance of a previously developed automated microtubule end tracking routine; this has been optimized for comparatively low signal‐to‐noise image sequences that are characteristic of microscopy movies of dynamic microtubules growing *in vitro*. Sequences of simulated microtubule images were generated assuming a variety of different experimental conditions. The simulated movies were then tracked and the tracking errors were characterized. We found that the growth characteristics of the microtubules within realistic ranges had a negligible effect on the tracking precision. The fluorophore labelling density, the pixel size of the images, and the exposure times were found to be important parameters limiting the tracking precision which could be explained using concepts of single molecule localization microscopy. The signal‐to‐noise ratio was found to be a good single predictor of the tracking precision: typical experimental signal‐to‐noise ratios lead to tracking precisions in the range of tens of nanometres, making the tracking program described here a useful tool for dynamic microtubule end tracking with close to molecular precision.

## Lay Description

Microtubules form part of the ‘skeleton’ of a cell and are highly dynamic, tubular polymers which grow and shrink in order to perform specific roles at different points of the cell's life cycle. A powerful method to study these microtubules is fluorescence microscopy; tracking the end position of microtubules in movies provides insight into the mechanisms that govern microtubule dynamics. It is important for further analysis of these dynamics that the position of the microtubule ends can be reliably and accurately determined throughout a microtubule's growth. Here we describe and analyse the performance of an automated microtubule end‐tracking program that has been optimised for microscopy image sequences of purified dynamic microtubules growing on a slide, outside the cellular environment. The errors associated with tracking the microtubule ends have been characterised here using simulated movies. Simulations allow a range of experimental parameters to be tested with knowledge of the true end position, to evaluate to the results of the tracking program. We found that the growth characteristics of the microtubules within realistic ranges had a negligible effect on the tracking precision. However, imaging conditions such as magnification and camera exposure time were found to be important parameters limiting the tracking precision. Simulations of typical experimental imaging conditions indicate microtubule ends can be found with a precision at the scale of the size of individual microtubule sub‐units; hence, the tracking program described here is a useful tool for studying subtle structural changes in the microtubule during growth.

## Introduction

Microtubules are dynamic intracellular filaments that are essential for a large variety of key processes in eukaryotic cells, such as cell division and differentiation. Associated proteins regulate microtubule dynamics or mediate microtubule interactions with other intracellular assemblies (Howard & Hyman, 2009; Duellberg *et al*., 2013). Fluorescence microscopy is often used to visualize microtubules and their associated proteins, either in living cells or in biochemical *in vitro* experiments. To better understand the molecular mechanisms giving rise to the dynamic properties of microtubules and underlying their regulation, it is important to be able to quantitatively measure the polymerization and depolymerization kinetics of individual microtubules with high precision, requiring automation (Danuser *et al*., 2000).

**Figure 1:**
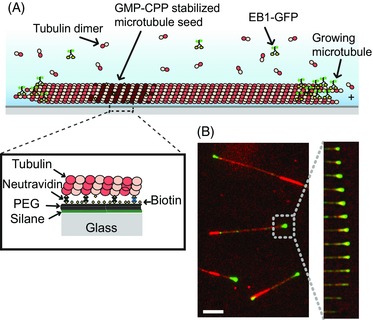
Experimental dynamic microtubule assay. (A) Schematic of a dynamic microtubule attached to a glass cover slip, showing a fluorescently labelled end binding protein, such as EB1‐GFP, accumulating at the growing microtubule end. (B) Left – Example image from a TIRF microscopy movie showing EB1‐GFP (green) binding to a Cy5 labelled microtubule (red). Right – time series of successive frames of the movie showing growth and shrinkage of a microtubule end.

An individual microtubule consists of tubulin‐heterodimers of about 8 nm length that arrange in 13 protofilaments to form a tube with an outer diameter of 25 nm (Fig. 1A) (Mandelkow *et al*., 1986); microtubules vary in length typically up to many micrometres. For fluorescence microscopy imaging, often around 10% of tubulins are labelled (on average 1 fluorophore ∼ every 6 nm along the microtubule axis). During imaging, the spatial distribution of the fluorophores is convolved with the microscope point spread function (PSF), with a full width half maximum (FWHM) of the order of ∼300 nm for conventional fluorescence microscopy. Additionally, for camera‐based imaging the captured image is pixelated due to the detector elements whose size corresponds to around 100 nm in sample space.

Despite these limitations, methods have been developed that allow tracking of microtubule ends with sub‐pixel precision. For time‐lapse movies of living cells, automatic tracking routines have been developed that track fluorescently labelled end binding proteins of the EB family which naturally accumulate at growing microtubule end regions and typically offer better contrast than labelled microtubules, thereby facilitating end recognition (Matov *et al*., 2010). Such methods are powerful for gaining a global overview of microtubule dynamics in living cells.

For several applications, however, it is necessary to track the microtubule ends themselves, especially when the microtubule end position needs to be known precisely during the entire microtubule life history. In this case EBs cannot be used as markers, as they only accumulate at growing ends, but not at pausing or shrinking microtubule ends (Fig. 1B). Furthermore, when the distribution of end binding proteins with respect to the microtubule end position is studied, information about both the microtubule end position and the binding protein needs to be collected independently. Such analysis has demonstrated that at the nanometre scale, differences exist between the localizations of different end binding proteins (Maurer *et al*., 2014). Finally, information about the shape of the ends of growing microtubules, i.e. the length distribution of the 13 individual protofilaments of a microtubule, can be of interest. Such information is typically obtained from electron microscopy images (Chretien *et al*., 1995), but in principle could be also extracted from fluorescence microscopy images (Gardner *et al*., 2011; Coombes *et al*., 2013; Maurer *et al*., 2014). Information about this protofilament length distribution, often called ‘end taper’ or ‘sheet structure’, could give further insight into the mechanism of growth and the mechanism underlying the transition to shrinkage.

Direct tracking of microtubule ends (in contrast to tracking end binding proteins) has been achieved previously in several ways (Demchouk *et al*., 2011; Ruhnow *et al*., 2011; Maurer *et al*., 2014; Prahl *et al*., 2014). One of the most prominent solutions relied on first determining the microtubule orientation in the image and then fitting a one‐dimensional model to the one‐dimensional intensity profiles along the microtubule axis (Demchouk *et al*., 2011; Prahl *et al*., 2014). This model considers PSF convolution and potential end structure effects, but does not consider the entire two‐dimensional information of a microtubule in the image when performing the fit. A subsequent method was developed where a two‐dimensional model was directly fitted to the image data [FIESTA (Ruhnow *et al*., 2011)], overcoming previous limitations. This method was developed for experimental data with a comparatively high signal‐to‐noise ratio (SNR): these were experiments where stabilized (static) microtubules were observed as they were transported over motor‐covered surfaces (gliding assays) or that were slowly depolymerized by depolymerases. In such experiments only negligible amounts of labelled free tubulin are in solution.

However, when dynamically growing and shrinking microtubules are imaged, high concentrations of free tubulin are typically present that considerably increase the background fluorescence (as both microtubule‐incorporated and nonincorporated tubulins are labelled at the same ratio). This leads to low SNRs, in which regime standard threshold‐based algorithms do not succeed at segmenting microtubules with high fidelity, even with considerable human input. Furthermore, growth of dynamic microtubules can be considerably more irregular than transport in gliding assays and transitions from growth to rapid shrinkage (and the reverse) need to be followed accurately.

Recently, we described an extension of the method of two‐dimensional model fitting with the aim to overcome these limitations encountered when tracking dynamic microtubule ends in movies with lower SNRs (Maurer *et al*., 2014). Several new elements were introduced to improve the tracking efficiency under these conditions, such as additional image filtering and microtubule growth/shrinkage predictions to input more reliable initial values into the two‐dimensional model fits of the raw data. This improved procedure for microtubule dynamics analysis (called MDA here) allows the automated generation of unbroken, long tracks of the end position of dynamically growing microtubules at low SNR with sub‐pixel precision. We had previously used the tracking programme for one specific experimental condition and characterized the precision of the procedure, using a bootstrapping approach (Maurer *et al*., 2014).

Here, we describe the features of the tracking programme that was designed to improve performance at lower SNR in much greater detail and quantitatively characterize the tracking performance for a wide range of image qualities, using simulated image data. We simulate changes in certain key imaging parameters and describe the resulting effects on the tracking precision; this enables us to suggest ways to optimize the data acquisition process for better analytical results. The tracking precision determines the ultimate quality of subsequent image analysis steps that rely on the found microtubule end positions, for example averaging profiles aligned with respect to the microtubule end position. We find that the precision of microtubule end localization is determined by the same principles that also determine point localizations. Furthermore, the tracking precision achieved can be estimated largely based on just considering the SNR: we find that for most combinations of realistic experimental parameters that give SNRs of 2 or higher, we can achieve microtubule end tracking with a precision in the range of tens of nanometres.

## Materials and methods

### Signal‐to‐noise ratio (SNR)

The SNR is an important parameter in this study. Since there is no universally accepted definition for the SNR of a microtubule in an image, we define it here using two 1 μm × 0.4 μm boxes, one placed along the backbone of the microtubule near its end, and one shifted 1 μm away from the microtubule to identify the background statistics (the width of the box is approximately the same as the apparent microtubule width in the image). The SNR is thence defined as

I MT −I BK /σ MT 2+σ BK 2,

where I_MT_ and σ_MT_ are the mean and standard deviation of the pixel intensities in the box centred on the microtubule, and I_BK_ and σ_BK_ are the mean and standard deviation of the pixel intensities in the background box.

### Experimental data

The tracking software is optimized for total internal reflection fluorescence (TIRF) microscopy movies of fluorescently labelled microtubules growing *in vitro* from surface‐immobilized stabilized microtubule seeds (Maurer *et al*., 2014) (Fig. 1A). The density of the more brightly labelled seeds is adjusted such that microtubules only infrequently cross each other. Although any fluorophore can be used to label tubulin, often fluorophores emitting at long wavelengths are used in order to allow for simultaneous imaging of microtubule‐binding proteins tagged with, for example green fluorescent protein (GFP). Typical labelling ratios are in the range of 0.1–0.2 fluorophores per tubulin subunit and typical velocities for microtubule growth and shrinkage *in vitro* are in the range of 10–150 nm/s and 0.3–1 μm/s, respectively. Images are acquired every 0.2–3 s using an EMCCD camera with an effective pixel size of 120 nm for a 100× objective lens, and an exposure time of 100–300 ms. The sigma value of the typical optical PSF for fluorophores emitting around 670 nm was found to be ∼ 135 nm (FWHM ∼ 315 nm), measured using 100 nm fluorescent beads (Maurer *et al*., 2014). An example for a typical image and a time sequence of a growing Cy5‐labelled microtubule in the presence of the GFP‐labelled end binding protein EB1 is shown in Figure 1B (conditions as in Maurer *et al*., 2014). Due to the presence of micromolar concentrations of unpolymerized fluorescent tubulin, the mean SNR is quite moderate in the range of 2–3.

### Simulated data

The simulation of time‐lapse fluorescence microscopy data of dynamic microtubules was performed in MATLAB (The MathWorks, Inc., Natick, MA, USA) using a modified ‘model‐convolution’ approach (Demchouk *et al*., 2011).

**Figure 2:**
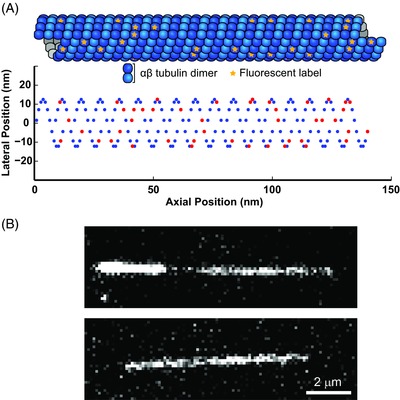
Simulation of a microtubule image. (A) Schematic representation of the 3D structure of a microtubule illustrating the random labelling of subunits (top) and the projection onto a 2D plane such that the position can be mapped into an image (bottom). (B) An experimental TIRF microscopy (top) and a simulated (bottom) image of a microtubule. (Note: the bright part of the experimental microtubule is the more brightly labelled ‘seed’.)

(1) For each image frame, the total number of subunits added to or lost from a 13 protofilament microtubule in the time since the previous frame was determined. For this number a normal distribution was assumed with mean defined by the average growth speed and variance defined by the fluctuations in growth speed (Oosawa, 1970; Gardner *et al*., 2011). To simulate a tapered microtubule end structure a linear distribution of protofilament lengths was maintained as subunits were added. (2) New subunits were randomly attributed a labelled or unlabelled state with a probability equal to the labelling ratio. (3) The [x, y] coordinates of the labelled subunits were then determined for each frame (using the 3 start B lattice model with radius 25 nm and α‐β tubulin subunit length 8 nm (Mandelkow *et al*., 1986) (Fig. 2A). To account for thermal bending fluctuations, the position perpendicular to the microtubule axis was then modified in each frame using a 1st order, uniformly loaded, cantilever beam model (Gittes *et al*., 1993) with the seed considered clamped and a normally distributed random tip deflection. The axial subunit positions were not modified since deflections are small relative to the microtubule length; we calculated the maximum axial position error contribution as a result of this assumption to be in the order of 0.1nm. The entire coordinate set was rotated and translated to position the microtubule in the desired location within the image frame.

The final steps of the simulation render the image sequence by model‐convolution using pixel centre, point sampling of a 2D Gaussian with sigma of 135 nm, as an approximation to the microscope PSF. (4) An image representing the mean, expected photon count detected in each pixel was constructed by calculating the sum of the contributions of all 2D Gaussians centred on each lattice bound labelled subunit position added to a uniform background photon count contributed by free labelled tubulin. This provides an instantaneous snapshot of a single state of the microtubule. (5) To account for the effects of microtubule growth during a finite exposure time, the peak intensity of the Gaussian contribution from subunits added or removed during the exposure time was adjusted assuming that the time spent in the lattice was linearly distributed. This provided the noiseless images for the purpose of infinite SNR simulations (data in Fig. 6). (6) To simulate noise the number of photon arrivals at each pixel was sampled from a Poisson distribution with the variance equal to the mean photon count previously calculated. This value was multiplied by the camera gain and added to the empirically determined constant offset representing the EMCCD read noise to produce the final simulated images (Fig. 2B) (data in Fig. 7) (Hirsch *et al*., 2013). In our experimental setup, variation in the read noise of the EMCCD camera was insignificant in comparison to the Poisson noise associated with the stochastic photon arrival statistics.

### Tracking

**Figure 3:**
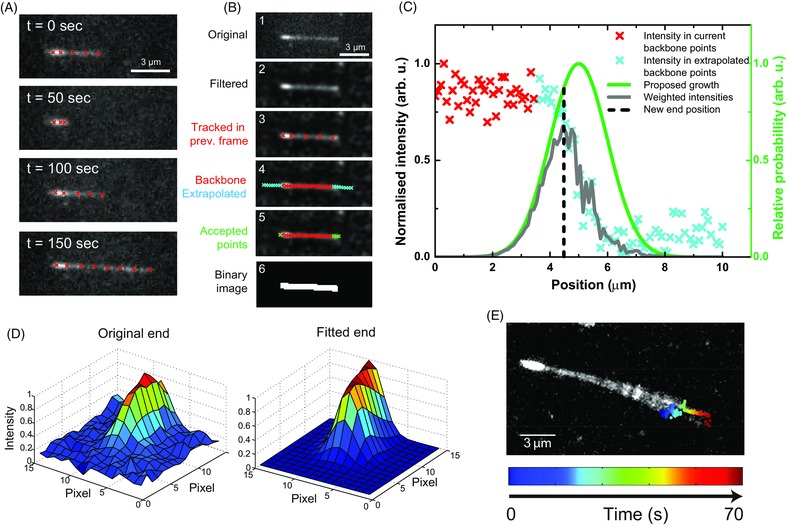
Microtubule end tracking. (A) Time sequence of TIRF microscopy images of an experimental dynamic microtubule tracked over a long period; the number of fitted red points varies to follow changes in total microtubule length. (B) Illustration of the steps performed to identify pixels belonging to a microtubule in the current frame (see main text). (C) Schematic showing how a new end point is initially determined in the current frame, using extrapolated points based on the current backbone positions. A proposed growth function is used to weight the 1D intensity profile, based on experimental parameters and the current state of the predictive dynamic model. (D) A 2D least squares fit (right) to the original, unfiltered experimental data (left) is used to determine the microtubule end. The same fitting procedure, with a configurable parametric model, is carried out for each fitted point. (E) Example maximum intensity projection of a wiggling microtubule during a growth period; the coloured points show the end position found from the 2D fitting at each time point. This illustrates that a ‘wiggling’ microtubule end can be tracked reliably.

The goal of microtubule end tracking is to identify the end position of growing or shrinking microtubules within each image frame of a movie with highest possible accuracy and reliability (Fig. 3A). To address the challenges originating from relatively high background, i.e. relatively low SNR, as well as large frame‐to‐frame end displacements due to low frame rates, typical for *in vitro* experiments with dynamically growing and shrinking microtubules, we have modified the pipeline of a previously published tracking software FIESTA (Ruhnow *et al*., 2011). The principal aim of the improved procedure that we call here MDA was to ensure that noise which generates apparent gaps within the microtubules does not result in false end detections and that, despite the considerable noise, reliable initial values are provided to the two‐dimensional fitting routine in every frame which is critical for maintaining the track. To achieve this we use the following workflow:

- Rough microtubule identification

Microtubules to be tracked are first roughly identified in one image of a movie by the user who mouse‐clicks along the microtubule contour (Fig. 3A, *t* = 0). All subsequent steps are automated (see GUI in Fig. S1) and are highly configurable (see Table S1). The first operation of the programme in every image frame is to identify all pixels that belong to each user‐marked microtubule (Fig. 3B). To this end, every frame is filtered with a Wiener filter to reduce noise, and subsequently with a Wallis filter (Wallis, 1976) to remove changes in intensity and contrast that vary slowly across the field of view (Fig. 3B, second image). This corrects for spatial variations of the excitation intensity in different parts of the image and for temporal variations of the emission intensity, for example due to bleaching, and ensures that the signal values are comparable over space and time.

To identify the pixels belonging to a specific microtubule, the program performs the following steps for each movie frame.

1. The image is smoothed using a configurable averaging filter. The backbone of the microtubule is found in the current smoothed image by identifying the brightest pixels in the vicinity of the backbone points of the microtubule as they were identified (or user‐provided) in the previous image frame (Fig. 3B, third image, red crosses), allowing only perpendicular positional variations with respect to the previous axis along the densely interpolated backbone points; this enables tracking of microtubules whose backbone moves in the image sequence either due to image drift or thermal motion (wiggling) (Fig. 3E).
2. A one‐dimensional intensity profile along the axial direction of the microtubule is created that includes extrapolated points outside the microtubule in the direction of the backbone (Fig. 3B, fourth image).
3. As the position of the microtubule end can be more difficult to identify in some image frames than in others due to noise, the programme uses information from previous as well as subsequent image frames to avoid losing track of the microtubule end in the current frame: a simple dynamic model is used to keep track of whether the microtubule in a specific frame is polymerizing or depolymerizing, allowing for transitions between two frames. The dynamic state of the microtubule is changed from ‘polymerization’ to ‘depolymerization’ (and vice versa) only if the microtubule end cannot be detected in the vicinity of its expected position for several consecutive frames. This avoids losing tracks due to short‐term imaging artefacts. As both states are typically stable over a short time period, once a transition occurs, the algorithm does not allow rapid conversion back into the previous state.
4. Pixel intensities are measured in the current and extrapolated backbone coordinates (Fig. 3B, red and cyan crosses), producing a one‐dimensional intensity profile in the current frame (Fig. 3C, red and cyan crosses). An approximately Gaussian ‘proposed growth’ function is generated (Fig. 3C, green curve); this is based on the previous frames’ tracks, the state of the dynamic model, and the experimental parameters, most importantly the growth speed and variability. The width of the proposed growth function is set such that transient phases of depolymerization are allowed, even when the microtubule is in the ‘polymerization’ state. The raw intensity data is then weighted by the growth function (Fig. 3C, grey line) and the position of the maximum is defined as a new proposed end point for subsequent fitting (Fig. 3C, black dashed line).
5. The accepted pixels that belong to the microtubule along the backbone including the pixel containing the new predicted end position in the current frame (Fig. 3B, fifth image) are now represented in a binary image (Fig. 3B, sixth image). Morphological operations (dilation and closure) are used to create a connected and thicker binary image, corresponding to the full extent of the microtubule in the image. This binary image of the microtubule is passed on as an initial positional estimate for the subsequent two‐dimensional fitting procedure.

II Sub‐pixel precision two‐dimensional fit to the original image data

Two‐dimensional fitting is carried out on the original, nonfiltered image data, as implemented in the FIESTA software package (Ruhnow *et al*., 2011) (Fig. 3D). This method utilizes a standard gradient descent approach to fit a parametric model to the image data. The main parameters are the continuous position, the width of the instantaneous PSF (to allow for potential changes in microtubule end structure that might affect the effective axial PSF), the background intensity level, as well as the direction and the brightness of the microtubule.

Here, unless otherwise indicated, we use a Gaussian wall‐end model for the two‐dimensional fit to the microtubule image that assumes a blunt microtubule tip. The ‘measured’ microtubule end position corresponds to the half maximum of the Gaussian intensity along the microtubule axis. Below, we will compare this measured end position to the a priori end position of simulated microtubule movies, directly providing the tracking error. The tracking software also allows the use of more complex fitting models with more parameters, for example allowing explicit consideration of end taper effects along the microtubule axis by defining an additional variance parameter, which allows elongation of the Gaussian fit axially. However, we will show that such models tend to generate larger tracking errors for equivalent SNRs.

The final output of the tracking procedure is the measured nanometre precision end position for the microtubule end in each tracked frame, along with values and estimated errors for all other fitted model parameters. The reliability of the fit is also calculated, which can be used to decide whether the track should be included in further analysis.

## Results – analysis

**Figure 4:**
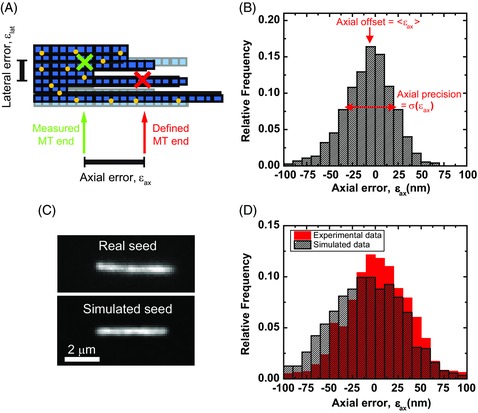
Localization errors from tracking simulated and experimental static microtubule ends. (A) Schematic of a simulated microtubule end showing tubulin dimers (blue squares) labelled with fluorophores (yellow circles). The simulated end position is defined as the mean of the coordinates of the final subunit on each of the 13 protofilaments, independent of labelling state (red cross). The difference between this position and that found from tracking the final movie (an arbitrary example position is shown as a green cross) define the axial and lateral localization errors. (B) Representative histogram of the axial errors found from tracking simulated microtubules. The offset and precision are defined as the mean and standard deviation of the errors, respectively. (C) A TIRF microscopy image of an experimental static GMPCPP‐stabilized microtubule (top) and a corresponding image of a simulated static microtubule (bottom). (D) As B, with the results of tracking 12 real microtubule seeds over 201 frames, and 15 simulated seeds, generated with similar imaging parameters to the real seeds, over 50 frames.

In order to quantitatively characterize the accuracy and precision of the MDA tracking algorithm under a range of experimental conditions, *in vitro* dynamic microtubule data was simulated and tracked as described in the previous section. We define the end position of the simulated microtubule as the mean of the coordinates of the final subunit on each of the 13 protofilaments, independent of labelling state at the end of the frame (Suppl. Note) (Fig. 4A). This provides the ‘ground truth’ against which the measured end position, determined by tracking, can be compared. For illustrative purposes we project the [x, y] localization error onto the axis of the microtubule, giving an axial and lateral error parallel and perpendicular, respectively, to the microtubule backbone (Fig. 4A). After tracking the simulated end over many frames, the distribution of the localization error has two principal descriptive components: the mean, which defines the accuracy, referred to here as the *offset* from the true end to be more explicit, and the standard deviation, which defines the *precision* (Fig. 4B).

Firstly, to validate the quality of the simulated data, we compared tracking results of simulated data to those of experimental data for the simplest case of stabilized seeds. This allowed the estimation of the errors of the tracking algorithm by performing an experimental control in the absence of free tubulin, and consequently in the absence of polymerization dynamics. We recorded a TIRF microscopy movie of surface‐immobilized GMPCPP‐stabilized microtubules in the absence of free tubulin, generating images with a high SNR of > 3 (Maurer *et al*., 2014) (Fig. 4C, top). A similar static microtubule was simulated with the same labelling ratio, intensity, and SNR (Fig. 4C, bottom). The distribution of the axial errors of the tracked microtubule end positions (Fig. 4D) defined a precision of 41 nm and 42 nm in the experimental and simulated cases respectively, indicating we can produce realistic estimates of the precision using simulated data. This validates the simulation, and using it we investigated the performance of the tracking algorithm for a variety of conditions, including those characterized by a low SNR.

**Figure 5:**
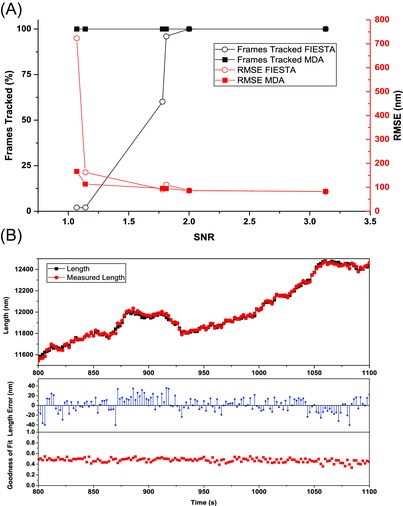
Comparison of MDA and FIESTA. (A) Comparison of tacking efficiency (frames tracked) and tracking errors (root mean square error in position) at a range of experimental SNRs. Parameters were optimized separately for both programs and manual thresholding was performed when using FIESTA to improve tracking efficiency. 100% of frames were tracked using MDA with at least as high precision as FIESTA at all SNRs. (B) Top: Example traces for simulated and measured length as obtained from tracking a simulated microtubule with highly diffusive growth using MDA. SNR = 3.81, V_g_ = 10 nm s^−1^, D_p_ = 300 nm^2^ s^−1^. Bottom: Errors in length measurement and goodness of fit (coefficient of determination) of the microtubule growth episode above.

Next, we examined the reliability of microtubule end detection in simulated movies over a range of varying SNR, comparing our method (MDA) with FIESTA (Ruhnow *et al*., 2011) which served as the starting point for our development (Maurer *et al*., 2014). Both methods performed similarly well at SNR > 2: essentially all microtubules in all frames were successfully identified with very similar tracking error (Fig. 5A). However at SNR < 2 the percentage of image frames with successfully tracked microtubule ends decreased strongly with decreasing SNR, whereas MDA still allowed successful end tracking at a SNR as low as 1.2 (Fig. 5A). The tracking error of the successfully tracked ends was also reduced at very low SNR when using MDA. This is an important feature for producing continuous tracks of dynamic microtubules where the mean SNR over the course of a movie is typically in the range of 2–3 but variations in individual frames can result in a SNR as low as 1.2. Reliable tracking was also observed during irregular growth episodes that contained brief depolymerization phases (Fig. 5B top), during which the tracking error and the goodness of the fit remained essentially unchanged (Fig. 5B bottom). Therefore, MDA is well suited for the specific image conditions typical for fluorescence microscopy movies of dynamic microtubules.

Next, using simulated movies, we examined in detail the consequences a variety of parameters have on the tracking precision of MDA, over a range of conditions typically encountered in experiments. Our analysis was performed for an effective PSF of 135 nm, as previously determined experimentally for objects labelled with far‐red emitting fluorophores and imaged on a standard TIRF microscope (Maurer *et al*., 2014). Two subsets of simulation parameters were considered, which could contribute to the localization error. First, parameters intrinsic to the microtubule: the microtubule growth velocity v_g_; an effective axial diffusion of the microtubule tip position around its mean trajectory due to variance in the growth velocity, characterized by an effective diffusion coefficient D_p_; lateral deflection of the tip position due to thermal fluctuations, with standard deviation of deflection σ_LD_; and the taper length, TL, resulting from protofilament length differences.

**Figure 6:**
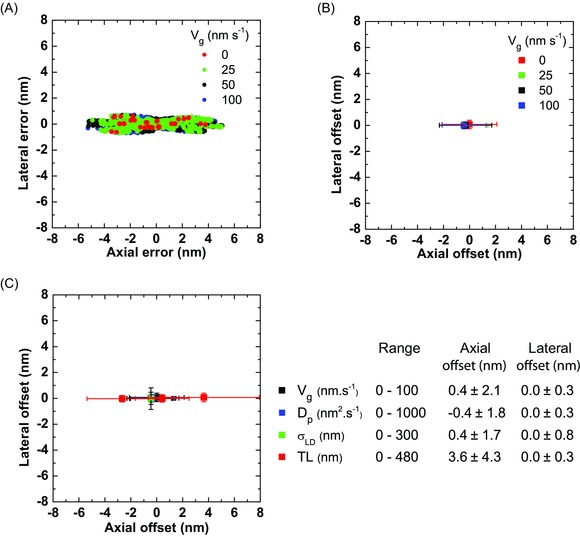
Localization errors from tracking simulated growing microtubule ends with different growth characteristics. (A) Scatter plot of individual localization errors of tracking simulated microtubules growing with different velocities, v_g_. (B) Average axial and lateral offset for each data set in (A). Error bars are associated precision values. (C) Average axial and lateral offset for simulated microtubules growing at 50 nm/s, with different end diffusion (D_p_), lateral displacement (σ_LD_), and taper length (TL). Error bars are associated precision values. Right: A summary of the results showing the largest error for each parameter range. For these simulations, the labelling ratio was kept at 1.0, the pixel size was 120 nm, and the exposure was instantaneous. A summary of all results is given in Table S2.

To study the effect of varying these parameters across a realistic range of values, data was first simulated with no noise and 100% labelling of tubulin dimers (Table S2). Simulated microtubules with mean growth velocities in the tested range from 0 to 100 nm/s were tracked very reliably. A scatter plot of axial versus lateral errors (Fig. 6A) and a more condensed presentation of the means (offsets) and standard deviations (precision) (Fig. 6B) shows that, independent of the growth velocity, the error distributions showed negligible offset and excellent precision with only ∼ 2 nm deviations for the axial and ∼0.3 nm deviations for the lateral error. The better precision for lateral versus axial microtubule end position determination is due to the stronger constraints for the fit perpendicular to the microtubule axis. Similar results were obtained when the effects of varied growth fluctuations, lateral wiggling, and taper lengths were examined (Fig. 6C). Parameters determining the movement of the tip, both axially and laterally, produced sub‐nanometre tracking offsets. The taper length was seen to affect tracking precision but not tracking offset. The maximum observed axial offset in no noise conditions was ∼ 4 nm for the largest tested taper length of 480 nm (Fig. 6C). Hence, in the absence of noise, tracking errors were negligible and minimally affected by variations in the microtubule growth characteristics, considering that microtubules are 25 nm wide and that single tubulin subunits are 8 nm long.

We then investigated the effect of intrinsic microtubule growth parameters at three realistic SNR values: We found that also under these lower SNR conditions, there were no large variations in tracking offset with respect to changes of the growth velocity (v_g_), the effective diffusion constant (D_p_), or the taper length (TL) (Fig. S2 A–C, Table S3). A deterioration in the axial tracking precision was only seen with increasing taper length (Pearson correlation coefficient was 0.37, two tailed p = 0.034). However, this change was much smaller than the observed changes due to changes in the SNR which had a dominating influence (Fig. S2 D).

We subsequently investigated the performance of a fit with a free parameter describing the axial standard deviation of a tapered microtubule tip, σ_end_ compared to a Gaussian wall‐end model (Fig. S3 A, B, Table S4) confirming that the precision deteriorates slightly at longer taper lengths (Demchouk *et al*., 2011) but that the tracking offset is minimally affected. We also find that this additional free fitting parameter results in a less precise end position determination as expected due to the additional degree of freedom. For a one‐dimensional end model, σ_end_ has recently been described as the Pythagorean sum of the PSF sigma and the standard deviation of the protofilament lengths, $\sigma_{end}=\sqrt{\sigma_{PSF}^{2}+\sigma_{PF}^{2}}$ (Demchouk *et al*., 2011). We confirm that this relation holds for the two‐dimensional end model (Fig. S3 C, D, Table S5). The notable increase in variability of σ_end_ for SNR < = 2 (Fig. S3 D) however indicates that at low SNR taper length measurements on individual frames were not reliable.

**Figure 7:**
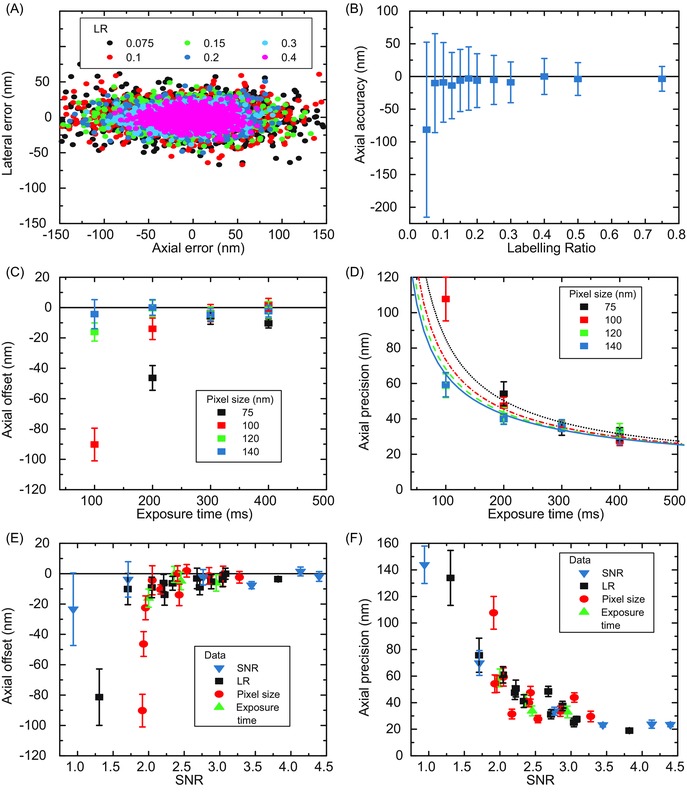
Localization errors from tracking simulated growing microtubule ends for different labelling ratios, exposure times and pixel sizes. (A) Scatter plot of individual localization errors from tracking simulated microtubules with different labelling ratios (LR), as indicated. (B) Average axial offset for each data set in (A). Error bars are associated precision values. (C) Average axial offset for tracked microtubules simulated with different exposure times and pixel sizes. Error bars are from bootstrapping. (D) Average axial precision for the data sets in (C). Error bars are from bootstrapping. Lines indicate global fits to the data using Equation 17 in Thompson *et al*. (2002), where the detected photon number is assumed to be directly proportional to the exposure time, and an additional EMCCD multiplicative noise factor of 1.4 is included. The optical background and readout noise were determined as 0 and 0.5 respectively from the fit. (E, F) Summary of data from (A)–(D) showing the average axial offset (E) and precision (F), for simulated microtubules: for each data set the average SNR value has been determined from the first frame of the movie. Error bars are from bootstrapping. The labelling ratio, the exposure time and the pixel size were 0.2, 200 ms and 120 nm, respectively, when they were not varied. The growth velocity (v_g_), the end diffusion constant (D_p_), the lateral displacement (σ_LD_) and the taper length (TL) were kept always constant at 11 nm/s, 271 nm^2^/s, 150 nm and 96 nm, respectively. A summary of all results is given in Table S6.

We subsequently investigated a subset of experimentally variable parameters that directly influences the SNR (Fig. 7, Table S6); this is known to be the principal determinant of precision in single molecule localization microscopy (Thompson *et al*., 2002; Shroff *et al*., 2008). Fluorescent microtubules are typically grown from mixtures of labelled and unlabelled tubulin so that ∼ every tenth tubulin in the microtubule is labelled (∼0.1 labelling ratio). Therefore, higher labelling ratios do not only increase the density of fluorophores on the microtubule, but also the background signal (tubulin in solution). Nevertheless the SNR will increase with higher labelling ratios, but this can also change the growth characteristics of the microtubules introducing artefacts in dynamic behaviour. For these reasons, we examined the effect of the labelling ratio on tracking performance using simulated microtubule movies. A scatter plot shows that lowering the labelling ratio clearly reduces the precision of tracking (Fig. 7A). We find, however, for a wide range of labelling ratios of 0.075 fluorophores per tubulin and greater, that the axial offset in the tracked end position is consistent and less than 10 nm (Fig. 7B). Despite the adverse effects on the signal variance as the labelling ratio is reduced, the axial precision initially deteriorates only very gradually (Fig. 7B), which can be rationalized by the concomitant decreasing contribution to the background. At a labelling ratio of 0.125 the axial precision is still 50 nm. The tracking performance only deteriorates precipitously at labelling ratios below 0.075, producing unacceptably large tracking offsets and low precision, because the density of the labels is too low for the tracking algorithm to reliably detect the feature defining the end position (Shroff *et al*., 2008). This information will be very useful for the experimental design.

In experimental data the SNR changes when the exposure time, the pixel size (e.g. by varying the magnification) or the illumination intensity are varied. Tracking simulated microtubules, with labelling ratio of 0.2 and imaged with exposure times varying from 100 ‐ 400 ms, shows a trend of decreasing tracking performance with decreasing exposure time (Figs. 7C,D, see also the Suppl. Note). This trend was also previously observed for mean square displacement analysis (Michalet, 2010). Concurrent variation of the pixel size from 75 nm to 140 nm has negligible effects for longer exposure times. However, the shortest tested exposure time of 100 ms requires pixel sizes of at least 120 nm. Nevertheless, for all pixel sizes the axial localization offset can be limited to below 15 nm (Fig. 7C) and the axial precision can be better than 40 nm (Fig. 7D) by choosing an appropriately long exposure time within the tested range. It is useful to note that at larger pixel sizes tracking performance would suffer due to undersampling, as demonstrated previously (Prahl *et al*., 2014).

Interestingly, we find that the dependencies of the precision on exposure time and pixel size can be well explained by models used for predicting precision in single molecule localization (Fig. 6D). We have used Equation 17 from Thompson *et al*. (2002) to perform a global fit of the data for each pixel size; the detected photon number is assumed to be directly proportional to the exposure time, and an additional EMCCD multiplicative noise factor of 1.4 has been included. This shows that, for nearly all choices of pixel size and exposure time, the tracking precision is limited by the fundamental imaging parameters rather than the microtubule dynamics or the software itself.

The parameters that affect the SNR were identified in our study as having the greatest influence on tracking performance; hence, we tested whether the SNR alone is sufficient to predict tracking performance. We found that the determined localization offsets (Fig. 7E) and the precision values (Fig. 7F) generated by varying either the labelling ratio, the pixel size, the exposure time or the signal intensity indeed fall onto a single curve when expressed as a function of the resulting SNR of the simulated images. We conclude (Figs. 7E,F) that a localization offset of less than 16 nm and a precision of better than 60 nm can be consistently achieved above an SNR of 2.0 and this can be improved to a performance of < 5 nm offset and < 40 nm precision at experimentally achievable SNR ratios of ∼3.

## Discussion

Here we examined in detail an automated tracking program (MDA), designed to identify and track dynamic microtubule ends with nanometre precision. Compared to a previous approach which served as the starting point for developing our method (Ruhnow *et al*., 2011), the major advance here is a largely improved tracking reliability at low SNR. This is a consequence of an improved estimation of the rough microtubule end position that serves as the start value for the final two‐dimensional fit to the original image data. This overcomes a limitation of the previous approach and allows now also reliable tracking of microtubule ends at SNR < 2 (down to ∼ 1.2). At higher SNR both methods perform similarly, because they share the procedure performing the final two‐dimensional fit to the original image data.

We have investigated the performance of our program at low and high SNR using simulated microtubule data, generated for a range of experimental parameters representative of typical TIRF microscopy time lapse movies. Such an approach was previously demonstrated to be valuable for the study of the localization precision in single molecule localization microscopy (Sinko *et al*., 2014).

For typical experimental conditions with respect to, for example, labelling ratio and exposure time that are known to allow for long‐term imaging of dynamic microtubules without artefacts, we find that

1. the offset between the simulated and tracked microtubule end position can be limited to ∼10 nm or less (approximately the size of a single tubulin subunit), with an associated precision in the range of several tens of nanometres,

2. the tracking performance is essentially independent of the microtubule growth properties, and only depends significantly on the SNR of the data: specifically, for SNR values above 2 the localization precision is completely consistent with that of a static single point emitter (Thompson *et al*., 2002; Shroff *et al*., 2008).

A variety of postprocessing data analyses have been possible due to the accuracy achievable with this tracking method. Two recent examples are as follows.

First, analysis of the distribution of the average binding site regions of microtubule end binding proteins with respect to the growing end: Once tracked, the microtubule end positions can be used as a reference for alignment of images of an associated end binding protein, simultaneously recorded in a different fluorescence channel. Given the negligible offset error, many thousands of such images can then be averaged, greatly increasing the SNR (Maurer *et al*., 2014).

The localization precision in the average image is the standard deviation of the sample mean after averaging, thus proportional to the square root of the quadratic sum of the tracking precision and the sigma of the PSF in the secondary fluorescence channel over the square root of the number of images $\left(SD_{\bar{x}}\propto \sqrt{\sigma_{\mathrm{track}}^{2}}+\sigma_{\mathrm{PSF}}^{2}/\sqrt{n}\right)$. Therefore, for typical experimental conditions giving a tracking precision of ∼40 nm, a localization precision of the order of a single subunit can be achieved in the average image by averaging over approximately 300 individual images. This can be easily obtained from just a few movies with several microtubules tracked in each movie. Using detailed model fitting on the final averaged images of different end binding proteins, detailed information can be extracted about the spatial distribution of their binding sites relative to the microtubule end position (Maurer *et al*., 2014).

Second, catastrophe analysis: Dynamic microtubules can be tracked before and during a catastrophe. By temporally aligning position traces with respect to an objectively defined catastrophe time point, the conformational changes in the microtubule end region can be investigated by observing the average kinetics of the decay of the amount of a conformational sensor before catastrophe (Maurer *et al*., 2012; Maurer *et al*., 2014).

In the future, more detailed information with good statistics on the instantaneous position of the microtubule end during growth and shrinkage will be vital for investigations into the origin of microtubule growth fluctuations (Gardner *et al*., 2011). Furthermore, it will be interesting to try to gain information on the detailed structure of the end of a microtubule and to investigate its relationship with the growth properties of the microtubule (Coombes *et al*., 2013). We have shown here that the measurements of taper length in individual images at low SNR can be highly variable, but accurate alignment and averaging may allow for more complex models to be compared to an averaged end structure in which case the smallest resolvable structure will be determined by the number of frames averaged and the tracking precision for the individual images. Hence, for these and other investigations, it is essential to know the expected precision for tracking the ends of dynamic microtubules in sequences of image with a certain characteristic SNR.

## Supporting information

Supporting Information
**Table S1**: Configurable parameters used by the MDA software in the tracking and analysis pipeline. (A) Basic information about the data set. (B) Filtering options for microtubule segmentation. (C) Configurable parameters for the image processing and the dynamic model used during segmentation. (D) Options for extracting statistics of intensity data, given the tracked positions. (E) Models and averaging options for fitting spatial intensity profiles. (F) Options for temporally aligning tracks based on common features.
**Table S2**: Parameter values used for the simulation of growing microtubules in the absence of noise, as analysed in Figure 6, and the resulting axial and lateral offset and precision values obtained after tracking using MDA.
**Table S3**: Parameters used for the simulated microtubules as analysed in Figure S2 and the resulting axial and lateral offset and precision values and their errors obtained after tracking by MDA.
**Table S4**: Parameters used for the simulated microtubules as analysed in Figure S3 A, B and the resulting axial and lateral offset and precision and σend obtained after tracking using MDA with a fixed and free end sigma.
**Table S5**: Parameters used for the simulated microtubules as analysed in Figure S3 C, D and the resulting axial and lateral offset and precision and σend values and their errors obtained after tracking using MDA.
**Table S6**: Parameter values used for the simulation of growing microtubules as analysed in Figure 7 and the resulting axial and lateral offset and precision values and their errors obtained after tracking using MDA.
**Fig. S1**: Graphical user interface of the MDA tracking program.
**Fig. S2**: Localization errors from tracking simulated microtubule ends with different growth characteristics. Axial precision for simulated microtubules at three SNR values with different (A) growth velocity (v_g_), (B) end diffusion (D_p_) and (C) taper length (TL), respectively. Error bars are standard errors calculated from bootstrapping. (D) Data from (A), (B) and (C) and Table S3. Errors are standard errors calculated from bootstrapping. An offset of 0.02 in the SNR has been added to each point for display of overlapping points. For these simulations, the labelling ratio was kept at 0.2, the pixel size was 120 nm and the exposure time was 200 ms. A summary of all results is given in Table S3.
**Fig. S3**: Localization errors and σ_end_ from tracking simulated growing microtubule ends for different taper lengths and SNRs. (A) Axial offset and (B) precision, respectively, for different taper lengths (TL) tracked with fixed and free σ_end_. SNR = 1.71. Differences in axial offset and precision were determined by paired sample *t*‐test and two‐sample *F*‐test respectively; *N* = 750; * *p* < 0.05, ** *p* < 0.01, *** *p* < 0.0001. A summary of results is given in Table S4. (C) σ_end_ for tracked microtubules simulated with different taper lengths and SNRs. Theoretical hyperbola $\sigma_{end}=\sqrt{\sigma_{\mathrm{PSF}}^{2}+\sigma_{\mathrm{PF}}^{2}}$ in black where σ_PSF_ is the sigma value of the point spread function and σ_PF_ is the standard deviation of protofilament lengths. (D) Data in (C) re‐plotted against SNR. SNR values offset by 0.03 per TL series for visualization of overlapping points. Error bars are standard deviation, *n* = 750. The labelling ratio, the exposure time and the pixel size were 0.2, 200 ms and 120 nm, respectively. The growth velocity, the effective diffusion coefficient and lateral displacement were 11 nm/s, 271 nm^2^/s and 150 nm, respectively. A summary of results is given in Table S5.

## References

[jmi12316-bib-0001] Chretien, D. , Fuller, S.D. & Karsenti, E. (1995) Structure of growing microtubule ends: two‐dimensional sheets close into tubes at variable rates. J. Cell Biol. 129, 1311–1328. 7775577 10.1083/jcb.129.5.1311 PMC2120473

[jmi12316-bib-0002] Coombes, C.E. , Yamamoto, A. , Kenzie, M.R. , Odde, D.J. & Gardner, M.K. (2013) Evolving tip structures can explain age‐dependent microtubule catastrophe. Curr. Biol. 23, 1342–1348. 23831290 10.1016/j.cub.2013.05.059 PMC3762219

[jmi12316-bib-0003] Danuser, G. , Tran, P.T. & Salmon, E.D. (2000) Tracking differential interference contrast diffraction line images with nanometre sensitivity. J. Microsc. 198, 34–53. 10781207 10.1046/j.1365-2818.2000.00678.x

[jmi12316-bib-0004] Demchouk, A.O. , Gardner, M.K. & Odde, D.J. (2011) Microtubule tip tracking and tip structures at the nanometer scale using digital fluorescence microscopy. Cell. Mol. Bioeng. 4, 192–204. 23002398 10.1007/s12195-010-0155-6 PMC3445660

[jmi12316-bib-0005] Duellberg, C. , Fourniol, F.J. , Maurer, S.P. , Roostalu, J. & Surrey, T. (2013) End‐binding proteins and Ase1/PRC1 define local functionality of structurally distinct parts of the microtubule cytoskeleton. Trends Cell Biol. 23, 54–63. 23103209 10.1016/j.tcb.2012.10.003

[jmi12316-bib-0006] Gardner, M.K. , Charlebois, B.D. , Janosi, I.M. , Howard, J. , Hunt, A.J. & Odde, D.J. (2011) Rapid microtubule self‐assembly kinetics. Cell 146, 582–592. 21854983 10.1016/j.cell.2011.06.053 PMC3171214

[jmi12316-bib-0007] Gittes, F. , Mickey, B. , Nettleton, J. & Howard, J. (1993) Flexural rigidity of microtubules and actin filaments measured from thermal fluctuations in shape. J. Cell Biol. 120, 923–934. 8432732 10.1083/jcb.120.4.923 PMC2200075

[jmi12316-bib-0008] Hirsch, M. , Wareham, R.J. , Martin‐Fernandez, M.L. , Hobson, M.P. & Rolfe, D.J. (2013) A stochastic model for electron multiplication charge‐coupled devices–from theory to practice. PLoS One 8, e53671. 23382848 10.1371/journal.pone.0053671 PMC3561409

[jmi12316-bib-0009] Howard, J. & Hyman, A.A. (2009) Growth, fluctuation and switching at microtubule plus ends. Nat. Rev. Mol. Cell Biol. 10, 569–574. 19513082 10.1038/nrm2713

[jmi12316-bib-0010] Mandelkow, E.M. , Schultheiss, R. , Rapp, R. , Muller, M. & Mandelkow, E. (1986) On the surface lattice of microtubules: helix starts, protofilament number, seam, and handedness. J. Cell Biol. 102, 1067–1073. 3949873 10.1083/jcb.102.3.1067 PMC2114131

[jmi12316-bib-0011] Matov, A. , Applegate, K. , Kumar, P. , Thoma, C. , Krek, W. , Danuser, G. & Wittmann, T. (2010) Analysis of microtubule dynamic instability using a plus‐end growth marker. Nat. Methods 7, 761–768. 20729842 10.1038/nmeth.1493 PMC3032800

[jmi12316-bib-0012] Maurer, S.P. , Cade, N.I. , Bohner, G. , Gustafsson, N. , Boutant, E. & Surrey, T. (2014) EB1 accelerates two conformational transitions important for microtubule maturation and dynamics. Curr. Biol. 24, 372–384. 24508171 10.1016/j.cub.2013.12.042 PMC3969257

[jmi12316-bib-0013] Maurer, S.P. , Fourniol, F.J. , Bohner, G. , Moores, C.A. & Surrey, T. (2012) EBs recognize a nucleotide‐dependent structural cap at growing microtubule ends. Cell 149, 371–382. 22500803 10.1016/j.cell.2012.02.049 PMC3368265

[jmi12316-bib-0014] Michalet, X. (2010) Mean square displacement analysis of single‐particle trajectories with localization error: Brownian motion in an isotropic medium. Physical Rev. E. 82, 041914. 10.1103/PhysRevE.82.041914 PMC3055791 21230320

[jmi12316-bib-0015] Oosawa, F. (1970) Size distribution of protein polymers. J. Theor. Biol. 27, 69–86. 5419909 10.1016/0022-5193(70)90129-3

[jmi12316-bib-0016] Prahl, L.S. , Castle, B.T. , Gardner, M.K. & Odde, D.J. (2014) Quantitative analysis of microtubule self‐assembly kinetics and tip structure. Methods Enzymol. 540, 35–52. 24630100 10.1016/B978-0-12-397924-7.00003-0

[jmi12316-bib-0017] Ruhnow, F. , Zwicker, D. & Diez, S. (2011) Tracking single particles and elongated filaments with nanometer precision. Biophys. J. 100, 2820–2828. 21641328 10.1016/j.bpj.2011.04.023 PMC3117161

[jmi12316-bib-0018] Shroff, H. , Galbraith, C.G. , Galbraith, J.A. & Betzig, E. (2008) Live‐cell photoactivated localization microscopy of nanoscale adhesion dynamics. Nat. Methods. 5, 417–423. 18408726 10.1038/nmeth.1202 PMC5225950

[jmi12316-bib-0019] Sinko, J. , Kakonyi, R. , Rees, E. , Metcalf, D. , Knight, A.E. , Kaminski, C.F. , Szabo, G. & Erdelyi, M. (2014) TestSTORM: simulator for optimizing sample labeling and image acquisition in localization based super‐resolution microscopy. Biomed. Opt. Express 5, 778–787. 24688813 10.1364/BOE.5.000778 PMC3959829

[jmi12316-bib-0020] Thompson, R.E. , Larson, D.R. & Webb, W.W. (2002) Precise nanometer localization analysis for individual fluorescent probes. Biophys. J. 82, 2775–2783. 11964263 10.1016/S0006-3495(02)75618-X PMC1302065

[jmi12316-bib-0021] Wallis, R. (1976) An approach to the space variant restoration and enhancement of images. Proc. Symp. on Current Mathematical Problems in Image Science. 10–12.

