# Supplementary material for: Important factors determining the nanoscale tracking precision of dynamic microtubule ends
Source: J Microsc. 2016 Jan 19;261(1):67–78. doi: 10.1111/jmi.12316 (PMC4832305; doi:10.1111/jmi.12316)
Supplement: Supplementary file 1 — Supporting Information Table S1: Configurable parameters used by the MDA software in the tracking and analysis pipeline. (A) Basic information about the data set. (B) Filtering options for microtubule segmentation. (C) Configurable parameters for the image processing and the dynamic model used during segmentation. (D) Options for extracting statistics of intensity data, given the tracked positions. (E) Models and averaging options for fitting spatial intensity profiles. (F) Options for temporally aligning tracks based on common features. Table S2: Parameter values used for the simulation of growing microtubules in the absence of noise, as analysed in Figure 6, and the resulting axial and lateral offset and precision values obtained after tracking using MDA. Table S3: Parameters used for the simulated microtubules as analysed in Figure S2 and the resulting axial and lateral offset and precision values and their errors obtained after tracking by MDA. Table S4: Parameters used for the simulated microtubules as analysed in Figure S3 A, B and the resulting axial and lateral offset and precision and σend obtained after tracking using MDA with a fixed and free end sigma. Table S5: Parameters used for the simulated microtubules as analysed in Figure S3 C, D and the resulting axial and lateral offset and precision and σend values and their errors obtained after tracking using MDA. Table S6: Parameter values used for the simulation of growing microtubules as analysed in Figure 7 and the resulting axial and lateral offset and precision values and their errors obtained after tracking using MDA. Fig. S1: Graphical user interface of the MDA tracking program. Fig. S2: Localization errors from tracking simulated microtubule ends with different growth characteristics. Axial precision for simulated microtubules at three SNR values with different (A) growth velocity (vg), (B) end diffusion (Dp) and (C) taper length (TL), respectively. Error bars are standard errors calculated from boo [file JMI-261-67-s001.pdf]

Supplementary Information: Important factors determining the nanoscale tracking precision of dynamic microtubule ends

Gergo Bohner, Nils Gustafsson, Nicholas I. Cade, Sebastian Maurer, Lewis D. Griffin, Thomas Surrey

Supplementary Tables

Parameter		Value (useful range)	Description
**(A) Basic settings**			
Config.workdir	=	'C:\MDA_default';	Working directory
Data.Input.General.PixelSize	=	120;	Pixel size
Data.Input.General.FPS	=	2;	Frames per second
Data.Input.General.MtStackNum	=	1;	Microtubule channel
Gui.general.scale	=	4/3; (0.6 – 4)	Scaling of the graphical user interface to accommodate different monitor resolutions - 1 corresponds to 1024*768.
Config.analysis.advanced.interpmethod	=	'cubic'; ('cubic', 'linear')	Interpolation method used anywhere required
Config.analysis.advanced.shownormalised	=	1; (0/1)	Show normalised or raw data anywhere required
**(B) Filtering settings**			
Config.settings.threshold.ThreshParams. Wiener.on	=	1; (0,1)	Automatic noise reduction for the approx. end identification
Config.settings.threshold.ThreshParams. Wallis.on	=	1; (0,1)	Apply Wallis filtering for the approx. end identification
Config.settings.threshold.ThreshParams. Wallis.args	=	{10000, 10000, 20, 1, 25, 1}; (1k-20k, 1k-20k, 1-50, 0-1, 5-50, 0/1)	{desired mean, desired standard deviation, maximum amplification, neighbour influence (0-1), window size(pixel), gaussian smoothing on/off}
**(C) Tracking settings for approximate end identification**			
Config.settings.mt_end_model	=	'e';	Type of end model fitted during tracking (character codes are in Model Library, but implemented models can be chosen in the GUI's drop down menu)
Config.settings.trackCatastrophes	=	1; (0/1)	Allow/Disallow tracking of shrinkage periods
Config.tracking.advanced.DynamicMarked. thresh_cat_value	=	-2500;	Intensity drop at the very end that initiates a check whether a catastrophe is happening or not
Config.settings.lookahead	=	4; (0-20)	Number of future frames to investigate for determining whether there is a real catastrophe or just an instantaneous intensity fluctuation (e.g. defocusing artefact)
Config.settings.threshold.ThreshParams. Threshold	=	1.2; (1.01 – 2)	If all backbone pixels that belonged to the microtubule are under background_mean * this threshold for the next lookahead frames, we initiate a catastrophe phase.
Config.tracking.advanced.DynamicMarked. neigh	=	2; (0-5)	Neighborhood for averaging filter to identify backbone
Config.tracking.advanced.DynamicMarked. avg_growth	=	2; (0-10)	in pixel, average expected growth speed
Config.tracking.advanced.DynamicMarked. lng	=	10; (1-30)	pixels; hard cap for maximum MT growth frame-to-frame
Config.tracking.advanced.DynamicMarked. variate_step	=	0.75; (0.25-2)	In pixels, step size perpendicular to the previous backbone to find the new maximum pixel laterally and thus counter the wiggling/drift.
Config.tracking.advanced.DynamicMarked. variate_num	=	4; (0-50)	# of sideway steps (increase in case of strong drift/wiggling)
Config.tracking.advanced.DynamicMarked. dseed_threshold	=	20; (0-40)	In pixel, end distance threshold from seed to counter seed brightness bias. We use two different distance thresholds to correct for the bias brightness gradually growing towards the seed.
Config.tracking.advanced.DynamicMarked. dseed_threshold_vclose	=	10; (0-40, < dseed_thresh)	

**(D) Extract raw data from secondary channel**			
Config.analysis.getgfpdata.cropsize	=	[6400 6400]; (500 – 20000)	nm, cropped area around tracked end for further analysis
Config.analysis.getgfpdata.croppixelsize	=	25; (10 – original pixel size)	nm, pixel size of the cropped area (sampling from the original pixel grid, but shifted and centered on the true microtubule end)
Config.analysis.getgfpdata.getintensities. boxsize	=	[1000 400];<br>(200 – 2000, ~PSF*(1-5))	[axial, lateral] nm, box used to determine statistics of intensity values around tracked end and at the lattice
Config.analysis.getgfpdata.getintensities. boxbehind	=	500; (0-2000)	nm, lattice box's start behind end's, to ensure there is no overlap
**(E) Averaging and fitting spatial profile**			
MtModels	=	ModelLibrary('MT_end');	
GfpModels	=	ModelLibrary('GFP_end');	
Config.analysis.averagegfp.models.mt	=	GetModelChar(MtModels{1});	Model used for MT averaged profile fitting
Config.analysis.averagegfp.models.gfp	=	GetModelChar(GfpModels{1});	Model used for GFP averaged profile fitting
Config.analysis.averagegfp.choose_align	=	0; (0/1)	Use channel alignment based on external information, if set to 1, it requires a static image of beads or ideally a grid, imaged in both channels)
Config.analysis.averagegfp.skipdarkgfp	=	0.15; (0 - 0.9)	Threshold for skipping frames with too low intensity (that are below skipdarkgfp * average gfp intensity)
Config.analysis.averagegfp.MT_cropsize	=	2000; (100 – cropsize)	nm, length used for fitting end models in the microtubule channel
Config.analysis.averagegfp.GFP_cropsize	=	4000; (100 – cropsize)	nm, length used for fitting models in the secondary channel
Config.analysis.averagegfp. NewAlignThresh	=	0; (- 100k – 10k)	nm , ensure that the microtubule has grown at least this much in the selected growth period, otherwise discard it
**(F) Investigating temporal evolution**			
Config.analysis.flexalign.mode	=	'Crosshair';	mode of alignment point determination:<br>- 'Timepoint' (same for all tracked microtubules, e.g. for flushing experiments)<br>- 'Crosshair' (user-determined catastrophe/rescue time point for each microtubule separately)<br>- 'Semiauto' (user-determined point of interest, but the catastrophe/rescue time point is determined by a parametrized linear fit to the microtubule length time series, see below)
Config.analysis.flexalign.params. timethreshold	=	4; (0.1 – 50)	sec, length of window checked by the semiautomatic checker around the user click.
Config.analysis.flexalign.slopefitlength	=	5; (1 – 30)	sec, width of averaging window for semiautomatic catastrophe/rescue point detection
Config.analysis.flexalign.params.slope	=	-250; (-50 - -1000)	nm/sec, minimum slope of linear fit to identify a catastrophe starting
Config.analysis.flexalign.params. regrowththreshold	=	20; (10 – 100)	nm/sec , minimum slope of linear fit to identify a rescue starting
Config.analysis.flexalign.subavgs	=	0; (0/1)	Use averages of subsets of the data (e.g. sorted by growth speed bins)
Config.analysis.flexalign.normalise	=	1; (0/1)	Individually normalise each track before alignment
Config.analysis.flexalign.avgfunc	=	'median';	{'mean'} or 'median', how to determine the average of multiple aligned tracks
Config.analysis.flexalign.params. laplacesmoothing	=	3000;	Parameter of Laplacian smoothing added to the normalization process to ensure no division by 0 due to artefacts/missed tracks etc.

Supplementary Table 1. Configurable parameters used by the MDA software in the tracking and analysis pipeline. (A) Basic information about the dataset. (B) Filtering options for microtubule segmentation. (C) Configurable parameters for the image processing and the dynamic model used during segmentation. (D) Options for extracting statistics of intensity data, given the tracked positions. (E) Models and averaging options for fitting spatial intensity profiles. (F) Options for temporally aligning tracks based on common features.

Experiment	V_g (nm/s)	D_p (nm²/s)	σ_{LD} of Lateral Deflection (nm)	Taper Length (nm)	Axial Offset (nm)	Axial Precision (nm)	Lateral Offset (nm)	Lateral Precision (nm)
**Growth Speed**	0.00	0.00	0	0	-0.51	2.11	0.07	0.33
	25.00	0.00	0	0	-0.44	1.86	-0.01	0.25
	50.00	0.00	0	0	-0.30	2.02	0.01	0.23
	100.00	0.00	0	0	-0.42	1.69	0.00	0.27
**Growth Fluctuation**	50.00	0.00	0	0	-0.30	2.02	0.01	0.23
	50.00	20.00	0	0	-0.30	1.45	0.00	0.33
	50.00	40.00	0	0	-0.56	1.44	0.03	0.22
	50.00	80.00	0	0	-0.33	1.65	0.02	0.26
	50.00	1000.00	0	0	-0.37	1.71	-0.01	0.28
	50.00	500.00	0	0	-0.30	1.79	0.03	0.28
**Lateral Deflection**	50.00	0.00	0	0	-0.30	2.02	0.01	0.23
	50.00	0.00	50	0	-0.45	1.76	-0.02	0.28
	50.00	0.00	150	0	-0.44	1.67	0.00	0.47
	50.00	0.00	300	0	-0.44	1.59	-0.02	0.83
**Taper Length**	0.00	0.00	0	0	-0.51	2.11	0.07	0.33
	50.00	0.00	0	96	-0.40	1.57	0.00	0.24
	50.00	0.00	0	192	-2.65	2.73	-0.03	0.25
	50.00	0.00	0	288	0.43	2.07	-0.01	0.29
	50.00	0.00	0	480	3.64	4.29	0.07	0.34
	50.00	0.00	0	672	10.35	7.83	0.37	0.46

Supplementary Table 2. Parameter values used for the simulation of growing microtubules in the absence of noise, as analysed in Fig. 6, and the resulting axial and lateral offset and precision values obtained after tracking using MDA.

SNR	Taper Length (nm)	Vg (nm/s)	Dp (nm^2/s)	Axial Offset (nm)	SE of Axial Offset (nm)	Lateral Offset (nm)	SE of Lateral Offset (nm)	Axial Precision (nm)	SE of Axial Precision (nm)	Lateral Precision (nm)	SE of Lateral Precision (nm)
1.66	0	10	300	-5.93	10.39	0.05	4.65	62.62	10.08	25.03	1.99
	96		20	-3.27	10.19	-0.07	2.86	73.06	6.98	23.37	2.66
			150	-6.44	9.23	0.96	2.93	61.32	6.69	23.97	2.39
			300	-8.75	9.63	1.05	3.22	68.98	15.77	23.44	2.41
		30	300	-4.66	11.39	-0.17	3.45	65.18	7.65	26.03	2.44
		50	20	-8.50	6.13	1.39	2.69	60.92	8.62	22.04	1.49
			300	-14.83	11.16	1.71	3.52	72.45	10.69	24.76	1.85
	288	10	20	0.80	13.67	-1.28	3.52	79.72	9.10	26.90	2.55
			300	-5.12	11.82	-1.63	3.23	77.18	8.97	23.95	2.77
		50	20	-7.76	13.51	1.47	3.39	77.44	11.36	25.20	2.16
			300	-7.96	12.46	0.27	4.32	76.88	8.30	25.81	2.96
3.23	0	10	300	3.38	2.97	-0.17	0.94	26.13	2.53	7.04	0.94
	96	10	20	1.86	3.53	0.31	1.05	24.74	2.07	7.98	1.13
			150	-2.29	3.85	0.33	1.26	27.66	3.81	7.77	1.05
			300	-1.95	3.48	0.08	1.08	26.17	2.09	7.16	0.68
		30	300	-3.56	2.99	-0.42	1.20	27.30	2.63	7.33	0.80
		50	20	-6.63	4.21	0.28	1.12	27.93	2.02	7.20	0.65
			300	-4.57	3.01	0.59	1.02	25.24	2.68	7.55	0.70
	288	10	20	1.64	4.77	0.38	0.76	31.62	2.26	7.24	0.62
			300	5.51	3.73	0.07	1.76	32.08	3.72	7.98	0.59
		50	20	-5.69	5.08	-0.19	1.18	34.48	2.96	8.17	0.86
			300	-3.65	3.92	-0.31	1.33	30.64	3.65	8.05	0.53
3.81	0	10	300	-1.74	3.09	0.03	0.69	22.94	2.03	4.66	0.44
	96	10	20	-3.39	2.28	0.10	0.74	18.04	1.62	4.81	0.31
			150	0.47	3.12	0.18	0.78	20.11	2.58	4.97	0.50
			300	-1.28	3.70	-0.06	0.82	21.55	2.31	4.75	0.54
		30	300	-3.91	3.77	0.08	0.51	23.23	2.52	4.88	0.45
		50	20	-5.85	3.52	-0.17	0.66	22.08	2.61	4.86	0.54
			300	-5.86	2.88	-0.31	0.53	22.83	2.40	4.73	0.52
	288	10	20	-1.10	3.73	-0.38	0.89	23.80	2.06	5.03	0.27
			300	4.69	4.23	0.16	0.46	27.48	4.87	4.94	0.54
		50	20	-4.61	3.61	0.44	0.80	27.39	4.14	4.81	0.43
			300	-2.97	3.66	-0.33	0.73	29.54	2.62	4.90	0.43

Constants: σ_{LD} 150 nm, labelling ratio 0.2, pixel size 120 nm, exposure time 200 ms.

Supplementary Table 3. Parameters used for the simulated microtubules as analysed in Fig. S2 and the resulting axial and lateral offset and precision values and their errors obtained after tracking by MDA.

Tracking Method	Taper Length (nm)	Axial Accuracy (nm)	Lateral Accuracy (nm)	Axial Precision (nm)	Lateral Precision (nm)	End Sigma (nm)	SD of End Sigma (nm)
**Fixed End Sigma**	192	-3.83	0.02	65.18	22.34	134.68	13.22
	288	-2.26	1.14	74.44	26.12	134.86	14.30
	480	-14.64	0.11	93.87	25.51	135.02	14.73
	672	-1.31	-0.79	118.52	27.22	135.91	16.19
**Free End Sigma**	192	-3.00	-0.55	71.29	30.90	151.71	104.00
	288	-4.44	1.22	95.19	34.14	167.70	117.47
	480	-18.88	-0.54	113.79	32.22	219.43	147.94
	672	-13.10	-1.84	150.64	33.97	277.84	239.19

Constants: SNR 1.71, σ_{LD} 150 nm, V_g 11.1 nm/s, D_p 271.2 nm^2/s, Labelling Ratio 0.2, Pixel Size 120nm, Exposure time 200ms.

Supplementary Table 4. Parameters used for the simulated microtubules as analysed in Fig. S3 A, B and the resulting axial and lateral offset and precision and σ_{end} obtained after tracking using MDA with a fixed and free end sigma.

SNR	Taper Length (nm)	Axial Accuracy (nm)	SE of Axial Accuracy (nm)	Lateral Accuracy (nm)	SE of Lateral Accuracy (nm)	Axial Precision (nm)	SE of Axial Precision (nm)	Lateral Precision (nm)	SE of Lateral Precision (nm)	End Sigma (nm)	SD of End Sigma (nm)
1.71	0	-11.08	11.22	-2.04	2.94	68.75	7.13	24.26	2.66	134.01	13.99
	192	-3.83	6.96	0.02	3.85	65.18	7.56	22.34	2.32	134.68	13.22
	288	-2.26	8.02	1.14	4.49	74.44	10.35	26.12	3.36	134.86	14.30
	480	-14.64	12.03	0.11	3.39	93.87	9.19	25.51	3.59	135.02	14.73
	672	-1.31	18.03	-0.79	3.47	118.52	11.49	27.22	1.49	135.91	16.19
2.77	0	3.94	3.93	0.42	2.64	32.84	3.93	12.75	1.25	135.57	41.91
	192	3.44	6.22	-0.36	2.04	33.80	2.48	13.50	1.27	150.08	43.62
	288	8.22	5.23	-0.70	2.40	35.85	3.72	13.63	1.94	165.37	42.94
	480	0.77	8.92	-0.43	1.82	63.11	7.64	13.33	1.21	216.14	66.60
	672	-17.30	7.93	0.22	2.11	57.69	8.12	14.20	1.79	262.35	63.67
3.94	0	6.04	2.09	0.64	0.78	21.06	3.19	5.47	0.46	135.29	19.81
	192	-3.82	2.99	-0.47	0.78	29.24	2.58	5.07	0.42	157.57	35.61
	288	-5.68	4.70	-0.25	0.59	28.38	3.15	5.45	0.63	175.27	33.84
	480	3.87	5.80	-0.50	0.86	31.52	3.07	5.21	0.50	213.15	34.84
	672	4.63	5.77	-0.74	0.59	53.58	4.01	5.16	0.50	272.10	51.84
4.40	0	0.17	4.10	-0.30	0.58	27.27	3.62	4.11	0.51	139.58	29.00
	192	4.36	3.10	0.03	0.62	27.64	2.50	3.99	0.42	149.04	28.29
	288	4.09	4.17	-0.13	0.53	28.65	1.82	4.34	0.51	164.29	27.48
	480	-2.13	4.59	-0.51	0.38	30.13	3.02	4.46	0.49	211.40	34.76
	672	5.34	4.76	-0.26	0.67	38.02	3.75	4.17	0.34	264.24	37.71
4.53	0	-0.78	3.35	0.12	0.38	20.25	2.88	3.19	0.34	138.26	17.78
	192	-2.46	2.44	0.12	0.59	20.14	2.11	3.34	0.45	148.51	19.16
	288	1.03	2.57	0.07	0.48	20.86	2.25	3.25	0.32	169.47	20.39
	480	0.36	2.93	-0.06	0.36	31.96	2.60	3.24	0.32	208.08	32.19
	672	-22.02	6.62	-0.27	0.35	42.11	4.20	2.99	0.36	288.29	44.87
4.94	0	2.56	1.70	-0.17	0.32	17.22	1.94	2.66	0.18	133.89	14.43
	192	3.42	2.72	-0.02	0.30	23.77	1.71	2.91	0.37	148.11	21.22
	288	1.87	4.56	0.15	0.37	35.19	6.61	2.74	0.38	169.81	29.20
	480	-2.22	3.07	-0.01	0.44	30.42	6.67	2.69	0.26	207.45	28.45
	672	4.60	12.67	-0.27	0.37	77.61	8.83	3.11	0.49	268.08	53.42
inf	0	6.73	2.87	0.03	0.07	25.25	9.15	0.44	0.30	136.12	13.50
	192	5.62	2.60	-0.11	0.04	18.49	11.10	0.38	0.21	149.37	9.91
	288	4.64	1.15	-0.13	0.02	8.12	8.09	0.12	0.07	164.57	3.41
	480	7.47	5.39	-0.13	0.09	32.13	11.78	0.60	0.26	209.37	11.15
	672	3.56	3.06	-0.32	0.03	18.81	15.87	0.32	0.18	268.03	9.70

Constants: σ_{LD} 150 nm, V_g 11.1 nm/s, D_p 271.2 nm^2/s, Labelling Ratio 0.2, Pixel Size 120nm, Exposure time 200ms.

Supplementary Table 5. Parameters used for the simulated microtubules as analysed in Fig. S3 C, D and the resulting axial and lateral offset and precision and σ_{end} values and their errors obtained after tracking using MDA.

Experiment	Labelling Ratio	Pixel Size (nm)	Exposure Time (ms)	Axial Offset (nm)	SE of Axial Offset (nm)	Axial Precision (nm)	SE of Axial Precision (nm)	Lateral Offset (nm)	SE of Lateral Offset (nm)	Lateral Precision (nm)	SE of Lateral Precision (nm)
Labelling Ratio	0.05	120	200	-81.4	18.6	134.0	20.7	-1.3	4.6	31.6	3.2
	0.075			-10.2	10.3	75.7	12.8	0.1	2.9	22.1	2.5
	0.1			-9.1	6.8	61.0	6.2	-0.2	2.1	19.3	2.2
	0.125			-13.9	6.9	50.7	6.3	-0.4	2.2	17.0	2.0
	0.15			-6.2	5.7	47.5	5.1	0.7	3.1	15.9	1.1
	0.175			-3.1	6.8	48.5	3.9	0.6	2.1	14.7	1.1
	0.2			-6.4	4.6	41.2	4.8	-0.7	1.8	14.0	1.6
	0.25			-5.3	4.7	37.5	3.5	0.1	1.6	12.2	1.0
	0.3			-9.0	4.9	31.2	3.4	0.1	1.7	10.6	1.2
	0.4			0.0	3.6	27.5	2.2	-0.7	1.8	10.0	1.0
	0.5			-3.9	4.7	25.1	3.2	-0.3	0.9	8.6	0.6
	0.75			-3.6	1.1	18.9	1.5	0.0	0.8	7.0	0.5
Pixel Size and Exposure Time	0.2	140	100	-4.3	9.6	59.3	6.9	-0.2	3.0	19.2	1.5
			200	0.1	5.1	39.9	3.0	-1.1	2.0	12.9	1.2
			300	-4.6	4.1	36.2	3.3	-0.3	1.4	10.3	0.9
			400	-2.4	3.8	29.6	4.0	0.3	1.3	8.7	1.1
		120	100	-16.1	6.0	58.7	6.7	1.3	1.8	20.4	1.8
			200	-0.4	5.3	41.8	4.6	0.2	2.2	13.9	1.3
			300	-4.8	5.8	33.8	3.7	-0.7	2.0	11.4	1.3
			400	-6.1	5.4	33.0	4.4	0.3	1.1	9.6	0.9
		100	100	-90.2	10.8	107.7	12.3	-0.8	2.1	29.5	1.5
			200	-14.0	7.1	47.5	4.8	0.1	1.1	14.8	1.7
			300	-3.6	5.6	35.8	2.7	-0.1	2.7	11.9	1.0
			400	1.9	4.1	27.7	2.8	-0.6	1.3	10.3	0.9
		75	100	Did not track							
			200	-46.4	8.1	54.2	6.7	0.5	2.8	16.9	1.6
			400	-10.4	3.0	31.4	3.7	-0.4	1.5	11.5	1.0
			300	-5.7	5.3	34.7	3.9	-0.4	2.3	13.5	1.4

Constants: V_g 11.1 nm/s, D_p 271.2 nm^2/s, σ_{LD} 150 nm, Taper length 96 nm

Supplementary Table 6. Parameter values used for the simulation of growing microtubules as analysed in Fig. 7 and the resulting axial and lateral offset and precision values and their errors obtained after tracking using MDA.

Supplementary Note

The ground truth end point of the simulated microtubule was defined as the mean end coordinate of the final subunit on each of the 13 protofilaments, independent of labelling state. The coordinates of the final subunit of any individual protofilament changes, within the exposure time of a frame, dependent on addition or loss of subunits. For simplicity and clarity the instantaneous coordinates of the final subunits at the end of the exposure time were used rather than the time averaged end position. Assuming 100% labelling this introduces a small additional error relative to the simulated image end point which takes account of changes at the end of the microtubule. This error contributes to the tracking offset in a velocity dependent manner and contributes to the tracking precision in a diffusion dependent manner.

The mean offset, $\langle dL \rangle$, introduced is given by

$$\langle dL \rangle = \frac{t_e \langle v_g \rangle}{2}$$

where t_e is the exposure time and $\langle v_g \rangle$ is the mean growth velocity.

The mean contribution to loss of precision σ_{dL} is given by

$$\sigma_{dL} = \sqrt{D_p t_e}$$

where D_p is the effective diffusion of the growth and t_e is the exposure time.

For representative parameters, $\langle v_g \rangle = 11$ nm s^{-1}, $D_p = 271$ nm^2 s^{-1} and $t_e = 0.2$ s the mean offset and reduction in precision contributed is ~-1.1 nm and ~7.4nm respectively. When a fractional labelling ratio is taken into account a smaller proportion of the subunits added or lost during the exposure time will contribute to differences between the simulated image and the defined end position. Consequently, this additional contribution to the errors is likely to be significantly reduced such as to be negligible in comparison to measured offset and precision under experimental conditions.

Supplementary Figures

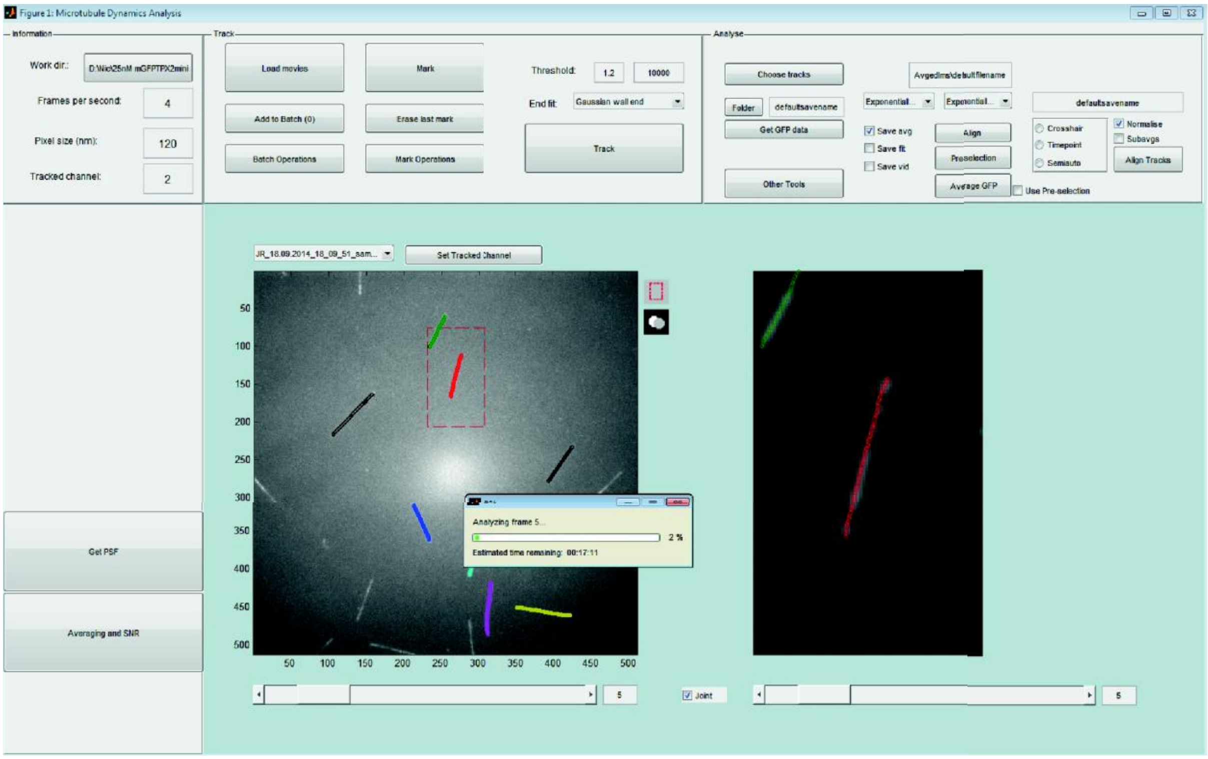

Supplementary Figure 1. **Graphical user interface of the MDA tracking program.**

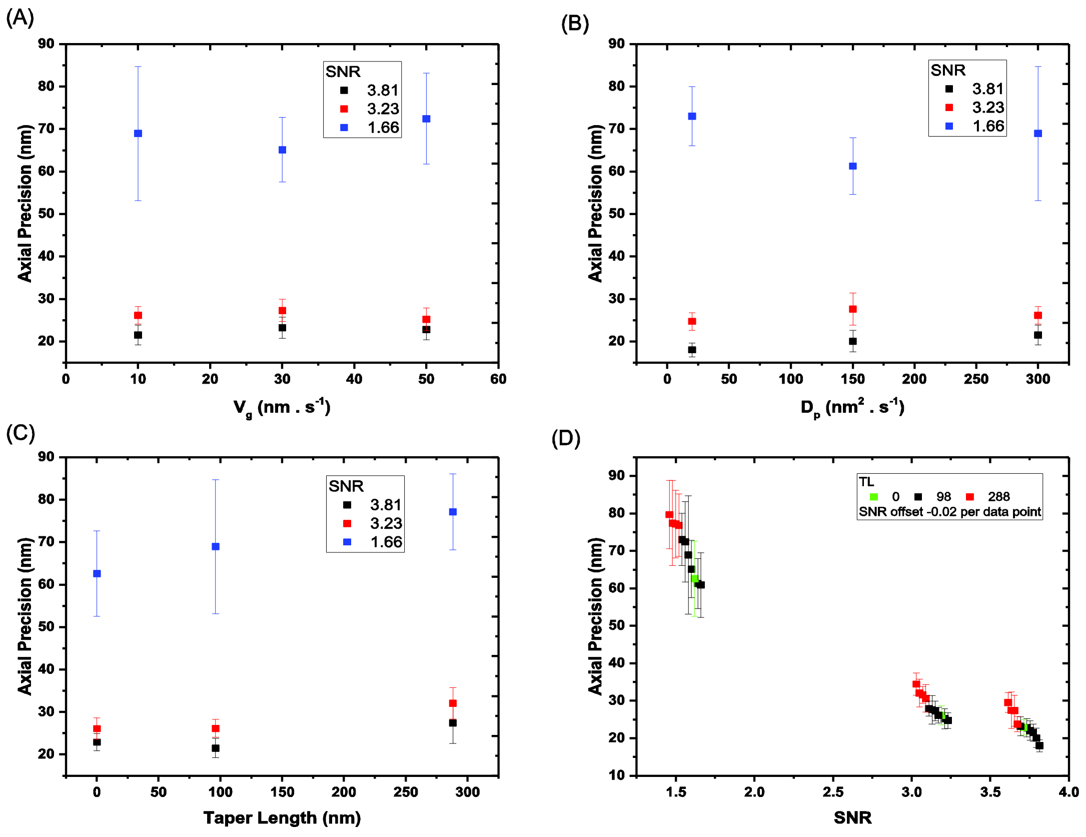

Supplementary Figure 2. Localisation errors from tracking simulated microtubule ends with different growth characteristics. Axial precision for simulated microtubules at three SNR values with different **(A)** growth velocity (V_g), **(B)** end diffusion (D_p) and **(C)** taper length (TL) respectively. Error bars are standard errors calculated from bootstrapping. **(D)** Data from (A), (B) and (C) and Table S3. Errors are standard errors calculated from bootstrapping. An offset of 0.02 in the SNR has been added to each point for display of overlapping points. For these simulations, the labelling ratio was kept at 0.2, the pixel size was 120 nm, and the exposure time was 200 ms. A summary of all results is given in Table S3.

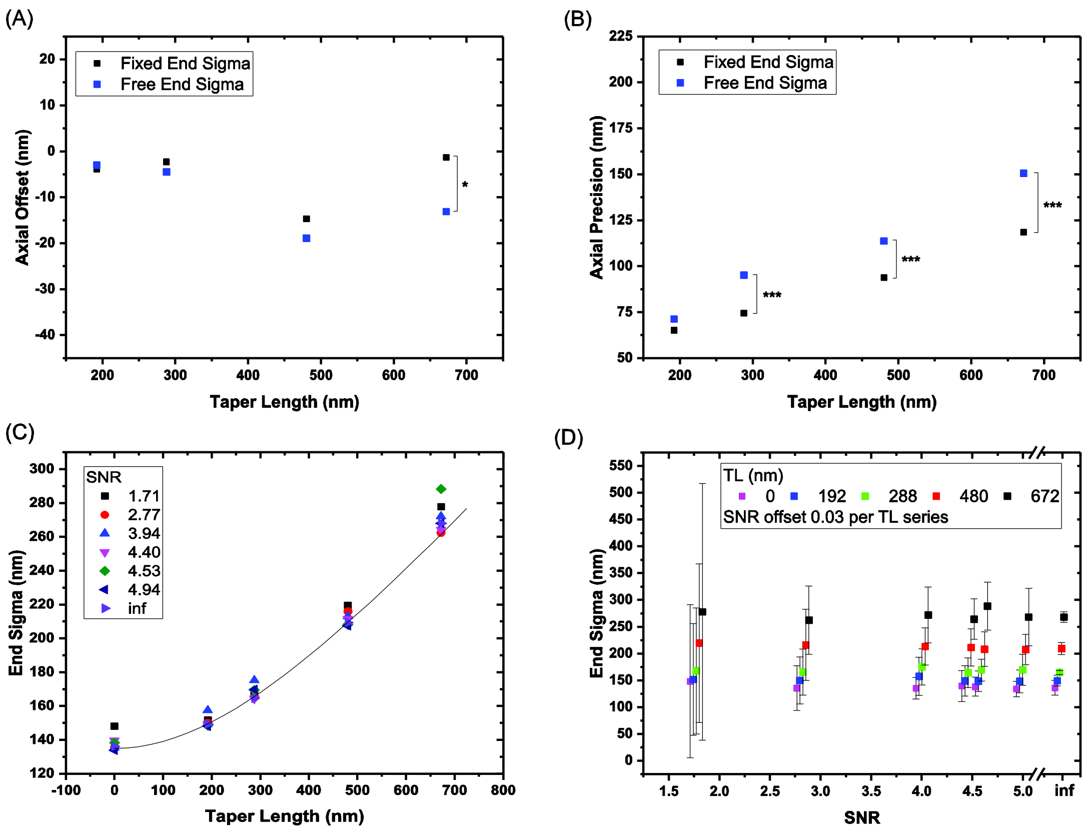

Supplementary Figure 3. Localisation errors and σ_{end} from tracking simulated growing microtubule ends for different taper lengths and SNRs. (A) Axial offset and **(B)** precision respectively for different taper lengths (TL) tracked with fixed and free σ_{end}. SNR = 1.71. Differences in axial offset and precision were determined by paired sample t-test and two-sample F-test respectively; N = 750; * $p < 0.05$, ** $p < 0.01$, *** $p < 0.0001$. A summary of results is given in Table S4. **(C)** σ_{end} for tracked microtubules simulated with different taper lengths and SNRs. Theoretical hyperbola $\sigma_{end} = \sqrt{\sigma_{PSF}^2 + \sigma_{PF}^2}$ in black where σ_{PSF} is the sigma value of the point spread function and σ_{PF} is the standard deviation of protofilament lengths. **(D)** Data in (C) re-plotted against SNR. SNR values offset by 0.03 per TL series for visualisation of overlapping points. Error bars are standard deviation, n=750. The labelling ratio, the exposure time, and the pixel size were 0.2, 200 ms, and 120 nm, respectively. The growth velocity, the effective diffusion coefficient and lateral displacement were 11 nm/s, 271 nm^2/s and 150 nm respectively. A summary of results is given in Table S5.
